# Abnormal brain functional networks in systemic lupus erythematosus: a graph theory, network-based statistic and machine learning study

**DOI:** 10.1093/braincomms/fcaf130

**Published:** 2025-04-02

**Authors:** Yifan Yang, Ru Bai, Shuang Liu, Shu Li, Ruotong Zhao, Xiangyu Wang, Yuqi Cheng, Jian Xu

**Affiliations:** Department of Rheumatology and Immunology, First Affiliated Hospital of Kunming Medical University, Kunming 650032, China; Department of Rheumatology and Immunology, First Affiliated Hospital of Kunming Medical University, Kunming 650032, China; Department of Rheumatology and Immunology, First Affiliated Hospital of Kunming Medical University, Kunming 650032, China; Department of Rheumatology and Immunology, First Affiliated Hospital of Kunming Medical University, Kunming 650032, China; Department of Rheumatology and Immunology, First Affiliated Hospital of Kunming Medical University, Kunming 650032, China; Department of Rheumatology and Immunology, First Affiliated Hospital of Kunming Medical University, Kunming 650032, China; Department of Psychiatry, First Affiliated Hospital of Kunming Medical University, Kunming 650032, China; Affiliated Mental Health Center & Hangzhou Seventh People’s Hospital, Zhejiang University School of Medicine, Hangzhou 310058, China; Department of Rheumatology and Immunology, First Affiliated Hospital of Kunming Medical University, Kunming 650032, China

**Keywords:** systemic lupus erythematosus, functional magnetic resonance imaging, brain functional network, network-based statistic, machine learning

## Abstract

Systemic lupus erythematosus patients’ brain functional network impairments are incompletely clarified. This study investigates the brain functional network topological alterations in systemic lupus erythematosus and the application of machine learning to the classification of systemic lupus erythematosus and healthy controls. Resting-state functional MRI data from 127 systemic lupus erythematosus patients and 102 healthy controls were used. The pre-processing process involved using automated anatomical labelling atlas to compute time series data for 116 brain regions. A functional connectivity network was then created by assessing the Pearson correlation between the time series of these brain regions. The GRETNA toolbox was used to compute the difference in topological attributes between groups. Variations in regional networks among groups were evaluated using non-parametric permutation tests that rely on network-based statistical analysis. With the functional connectivity network metrics as features and network-based statistical analysis as the feature selection method, network-based statistical analysis Predict software was used to classify systemic lupus erythematosus from controls by support vector machine. The subnets that contributed the most to systemic lupus erythematosus classification were also identified. For global indicators, the systemic lupus erythematosus group exhibited significantly lower values for the normalized clustering coefficient (*P* = 0. 0317) and small-world index (*P* = 0.0364) compared to the healthy controls group. After false discovery rate correction, the differences in Betweeness Centrality, Degree Centrality, Node Efficiency, Node Local Efficiency and other local indexes between the two groups were not retained. No correlation was found between clinical data and network indicators. Systemic lupus erythematosus group had a significantly reduced connection with a 12-node, 11-edge subnetwork (*P* = 0.024). In conclusion, systemic lupus erythematosus patients exhibit suboptimal global brain functional connectivity network topology and the presence of a subnetwork with abnormally reduced connectivity.

## Teaser

Yang *et al.* found suboptimal brain network organization and a subnetwork with low network connection in systemic lupus erythematosus patients using functional MRI. Functional MRI-based network analysis and support vector machine can identify systemic lupus erythematosus-related brain function abnormalities.

## Graphical Abstract

**Graphical Abstract:**
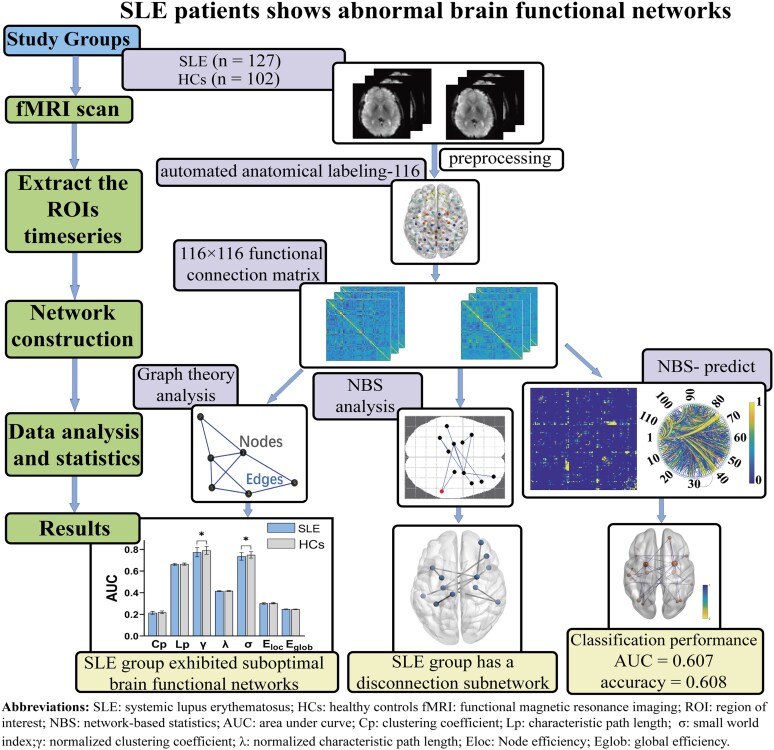

## Introduction

Patients with systemic lupus erythematosus (SLE) have an increased risk of cognitive impairment and mental disorders than healthy individuals.^1-3^ Studies using MRI have found that SLE patients have structural damage to both grey and white matter in the brain, resulting in reduced neurons, axons and white matter fibre bundles.^4,5^ This damage is believed to contribute to neuropsychiatric symptoms in SLE patients. However, brain function must be considered to fully understand the alterations in the brain associated with this complicated systemic disease.

Excitation-induced changes in neuron oxygen consumption and cerebral blood flow lead to alterations in local oxygenated and deoxygenated haemoglobin. Blood oxygen level-dependent functional magnetic resonance imaging (BOLD-fMRI) utilizes the ratio of oxygenated/deoxygenated haemoglobin as a contrast to non-invasively measure brain activity, showing potential for investigating brain alterations in complex disorders such as SLE.^6,7^ A multitude of studies has demonstrated impaired brain function in patients with SLE. A meta-analysis of 15 datasets, encompassing fMRI data from 572 SLE patients, revealed that brain dysfunction in SLE patients primarily manifested in the default mode network (DMN) and limbic system.^8^ Additionally, studies have shown that the degree centrality (DC) of certain brain regions, such as the bilateral hippocampus, were significantly altered in non-neuropsychiatric systemic lupus erythematosus (NPSLE) patients, reflecting changes in information integration ability.^9^ Another study focused on functional connectivity (FC) changes within the DMN in SLE patients, uncovering significantly reduced FC in five DMN nodes.^10^An independent component analysis study in SLE patients found increased FC in the right middle cingulate cortex, decreased FC in the left anterior cuneiform DMN and increased FC in the left cerebellar crus I and left posterior cingulate cortex.^11^A study analysing homotopic functional changes found that SLE patients had reduced bilateral hemispherical homotopic FC, and patients with NPSLE showed worse performance than patients with non-NPSLE.^12^ Previous fMRI study conducted by our team have also revealed anomalies in the intensity and stability of brain activity in SLE patients with cognitive impairment.^13^ Moreover, there is an interaction between the polymorphism of the serotonin transporter gene and SLE disease status on brain function.^14^ Abnormal brain FC has also been found to cause cognitive dysfunction in SLE patients by mediating disruption of the blood–brain barrier.^15^

Brain regions, clusters and neurons are linked by intricate structural and functional connections. This complex network processes and integrates information, enabling cognitive and behavioural tasks.^16^ Graph theory, an emerging network analysis method, can assess complicated brain network information integration and separation efficiency. Detecting functional linkages between brain regions, building a FC network and computing attributes to represent network features can indicate SLE patients’ possible brain function alterations at the network level.^17^ In addition, network-based statistics (NBS) also has the potential to provide meaningful insights into brain connectivity in various neurological diseases by identifying subsets of brain regions (subnetworks) containing abnormal interconnections within the whole-brain networks.^18^ Despite these advances, FC network studies related to SLE remain scarce.

A graph theory study estimated the topological features of whole-brain functional networks in SLE patients and found no global indicator difference between SLE and healthy controls (HCs) groups. Local indicators demonstrated lower nodal efficiency and degrees in the SLE group’s right insula, bilateral putamen, Heschl’s gyrus and right amygdala (AMYG).^19^ Another research found a lower clustering coefficient, global and local efficiency, and longer typical route length in non-NPSLE patients than HCs. Non-NPSLE patients had aberrant intra- and inter-network FC and reduced brain node strength. The research also found an inverse association between DMN intra-network FC and SLE disease activity index (SLEDAI).^20^ In NPSLE patients, the FC network had more modularity, with reduced connection in the bilateral hippocampus and right AMYG and increased connectivity in the left angular gyrus and left superior parietal lobules. Right hippocampal connection reduced while left angular gyrus and superior parietal lobe connectivity increased in non-NPSLE patients.^21^ A NBS study found that compared with HCs, patients with SLE had reduced FC within and between the DMN and CEN and increased connectivity within and between the sensorimotor networks (SMN).^22^ While this suggests a potential link between SLE and changes in FC networks, it is worth noting that there are inconsistencies in the current literature.

Machine learning (ML) techniques can classify patients and predict disease progress individually using MRI data. Recent research suggests its use in SLE. A multivariate pattern analysis classifier was created by Wang *et al*. using whole-brain DC data from 47 HCs and the same number with non-NPSLE. The accuracy, sensitivity and specificity of SLE categorization were 72.34, 63.83 and 80.85%.^9^ Despite these advances, FC network-based ML studies in SLE patients are lacking.

In summary, SLE has regional brain function anomalies, however, the brain functional network alterations need to be verified, and its usage in ML classification needs to be assessed. We are hypothesizing that SLE patients have brain functional network abnormalities that may affect cognition and mood regulations. The brain FC network parameters could be used as features for SLE classification. To test these hypotheses, this study will use graph theory, NBS and ML techniques to study SLE patients’ brain FC networks and conduct correlation analysis on clinical data and network attributes with differences to explore the brain network topological changes.

## Materials and methods

### Participants

Between 2008 and 2013, 140 SLE patients were recruited from the First Affiliated Hospital of Kunming Medical University. Additionally, during the same period, 111 healthy volunteers from the Health Management Center participated in the study as HCS. Each subject was interviewed by a clinically experienced rheumatologist and psychiatrist in the subject reception room dedicated to research before the MRI scan. The interview was conducted in detail and comprehensively, and the disease activity was assessed, with a focus on presence/absence of focal neurological signs and neuropsychiatric changes. The criteria for inclusion and exclusion are as follows:

#### SLE group inclusion criteria

Patients classified as SLE according to the 1997 American College of Rheumatology classification standard or the 2012 Systemic Lupus International Collaborating Clinics classification standard; No previous diagnosis of NPSLE, have not yet developed obvious neuropsychiatric symptoms, and had normal T1- and T2-weighted imaging scans on conventional brain MRI, that is, SLE patients without major neuropsychiatric manifestations; Age 18–50 years of age; Right-handed as determined by the Edinburgh Handed Inventory (scale score > 40 points).

#### HCs group inclusion criteria

Good physical health; Age 18–50 years old; Right-handed as determined by the Edinburgh Handed Inventory (scale score > 40 points).

Exclusion criteria of SLE and HCs group: meet the classification criteria of other connective tissue diseases, such as rheumatoid arthritis, Sjogren’s syndrome; Participants with organic brain diseases or neurological conditions that interfere with brain structure imaging, such as Parkinson’s disease, craniocerebral trauma, craniocerebral surgery history or epilepsy; Participants with severe psychotic manifestations, such as serious behavioural disorders and consciousness disorders; Participants have a history of psychoactive drugs, antipsychotic drug use history, drug, alcoholism and drug abuse; Participants have claustrophobia, metal implants and other MRI contraindications; Participants having a history of severe clinical diseases that might cause brain atrophy, such as hypertension, stroke and renal failure; Pregnant or lactating women; Conventional T1- and T2-weighted magnetic resonance plain scans indicated abnormal brain structure; Obvious head movement during scanning (translational > 2.0 mm, rotation > 2.0°).^23,24^

This study was approved by the First Affiliated Hospital of Kunming Medical University Ethics Committee. All participants and/or legal guardians were told of the experimental study’s objective and contents, volunteered to participate and gave informed consent before participation.

### Clinical data collection

In the study participants’ reception room, rheumatologists specializing in SLE management assessed disease activity in patients using the Systemic Lupus Erythematosus Activity Index 2000 and systematically examined for diffuse and focal neurological symptoms on the day of the MRI scan.^25^ The cognitive function, anxiety and depression in SLE patients were assessed by psychiatrists using the Mini-Mental State Examination (MMSE), the Hamilton Anxiety Scale (HAMA) and the Hamilton Depression Scale (HAMD), respectively.

### MRI data acquisition

#### MRI scanning method

The participants were instructed to avoid strenuous exercise and ensure adequate sleep from the day before the MRI examination. Inform the participants of the purpose and process of the examination, and instruct them to close eyes and rest, quiet breathing, relax and keep the body still during the scanning process and not carry out complex thinking activities. During the MRI scan, blood pressure, pulse and respiration are monitored and there is video monitoring in the scanning chamber and the subject is prompted at the end of each sequence to keep the participants awake.

#### MRI scanning parameters

MRI data were collected by an experienced radiologist using a 1.5T MRI scanner manufactured by General Electric (Twinspeed; GE Medical Systems, Milwaukee, WI, USA), complete with a birdcage head coil. The subject is placed in a supine position and a matching foam support pad is used to reduce the subject's head movement. Conventional T1WI and T2WI plain scans were conducted to rule out any apparent structural abnormalities.

##### sMRI data

The following parameters were used when adopting the 3D-T1 weighted fast spoiled gradient-echo sequence (3D-T1-FSPGR): echo time (TE) = 2.0 ms, repetition time (TR) = 10.5 ms, inversion time (TI) = 350 ms, layer thickness = 1.8 mm without layer interval, scanning matrix = 256 × 256, field of view (FOV) = 24cm × 18 cm, flip angle = 15°, spatial resolution = 0.94mm × 0.94mm × 0.9 mm, layers number = 172, a total of 846 s, the entire brain is covered by scans.

##### fMRI data

The following parameters are applicable to the gradient-echo sequence when using EPI technology: TE = 40 ms, TR = 2000 ms, number of excitation = 2.0, scanning matrix = 64 × 64, FOV = 24 cm × 24 cm, flip angle = 90°, layer number = 24, layer thickness = 5 mm, layer spacing = 1 mm, time point = 160 and total scan time is 320 s, covering the entire brain.

The MRI images of the study participants were carefully reviewed individually to confirm high quality and complete coverage of all brain tissue, including the brain and cerebellum.

### MRI data pre-processing process

DPARSF v4.4 was used based on the Matlab2016b platform to pre-process the BOLD-fMRI data.^26^ The specific data pre-processing process is as follows: (i) Remove the first 10 time points: to reduce the impact on the overall image due to the instability of the gradient magnetic field and the participant's inadaptability at the start of the scan; (ii) Time layer correction: fixing errors due by varied layer acquisition timings; (iii) Calculate and regress participants’ Friston-24 head motion parameters. To limit head motion’s impact on data quality and statistical results, translation and rotation >2.0 mm and 2.0° are excluded; (iv) Removal of the scalp, skull and other non-brain tissue; (v) Registering T1 structural images to fMRI; (vi) Segmentation: involves dividing the image into grey matter, white matter and CSF; (vii) Regress out noise signals: linear detrending, extraction and removal of covariates including CSF, white matter noise and Friston 24 head motion parameters; (viii) Filtering: select a filtering range of 0.01 ∼ 0.1 Hz to reduce noise outside the BOLD signal frequency; (ix) Spatial standardization and resampling: For latter comparison, each participant’s fMRI images were registered to the same template. Voxels in the present study were resampled to 3 × 3 × 3 mm^3^ using EPI templates based on Montreal Neurological Institute (MNI) space. (x) Smoothing: the full width at half maximum is ‘6, 6, 6’ to improve the signal-to-noise ratio. We also processed the sMRI data and calculated total intracranial volume (TIV), grey matter volume (GMV) and white matter volume (WMV) (see Supplementary materials for detailed procedures).

### Functional connectivity network construction

Based on the previous pre-processing results, a FC network was constructed using DPARSF.^27^ The brain was divided into 116 regions for this study using the automated anatomical labelling 116 (AAL-116) template. Each region becomes a network node. After computing the mean BOLD signal intensity of each brain region, the Pearson correlation coefficient between node pair average time series was calculated. Fisher-Z transformation normalized the Pearson correlation coefficient as the edge. Finally, the 116 × 116 FC matrix of each participant is created using the previously described nodes and edges. Since the functional connection of the brain region itself is not considered, the diagonal element of the matrix is set to 0.

### Function connection network analysis

#### Network analysis based on graph theory

In the Matlab2016b platform, GRETNA was used to analyse the functional connection network based on graph theory.^28^ GRETNA provides network configuration options. Sign: positive, negative, absolute; Threshold method: network sparsity and matrix element value; Network type: binary and weight. In the network formed by the brain FC matrix used in this study, there is a weight (−1, 1) relationship between all nodes and other nodes. There is currently no consistent explanation for the cases where weights are negative and close to zero^29^ so this study calculated absolute values. Participants utilized the same brain network template and had the same nodes. The brain network’s sparsity (the ratio of its actual edges to its maximum potential edges) is not the same since its edges are different. The network sparsity method was used to analyse brain network connection indicators to better explain negative and near-zero values, reduce sparsity’s impact on result indicators, and make network topological properties comparable between groups, based on previous relevant experimental experience.^30^ Binary and weighted networks have pros and cons in graph theory study. Binary networks simplify connections to presence or absence, making them easy to explain. Binarization can filter noise by setting thresholds to ensure that only strong connections are retained and reduce unnecessary complexity. Weighted networks capture network dynamics and small variances better due to connection strength. Our research examines functional connections rather than their strength. Thus, the binary network better represents our connection pattern. Due to our data and preparation, binarization enabled us to remove noise and ensure results reliability. According to relevant research, many studies used binary networks in comparable scenarios, confirming their efficacy.^31^ Therefore, we process the correlation matrix of each participant into an undirected binary matrix, whose sparsity range is fixed *D*_min_ – *D*_max_ range of *D*_min_ – *D*_max_.^32^ We also calculated the weighted network to examine the differences between the two network types, and the results are presented in the Supplementary materials. Referring to the highly recognized calculation method, the basis for selecting network density is defined as (i) the small-world index of all subjects’ networks >1.1 under each threshold; (ii) the network’s average node degree is >2 × log(*N*), where *N* represents the number of nodes in the network.^33^ In this study, *N* is 116. By calculation, the density of the network was set to 0.10–0.50 with a 0.02 interval. Based on to the sparsity threshold range, when the absolute value of the correlation coefficient between nodes *i* and *j* is greater than the threshold value, *aij* is 1; otherwise, it is set to 0.

#### Network indicator

The local properties of 116 nodes and the global properties of the brain network at each level of sparsity were computed respectively. A network’s global properties comprise a number of key metrics. The clustering coefficient (Cp) measures neighbourhood node connectivity. The normalized clustering coefficient (*γ*) measures the degree of connectivity between network nodes and their neighbouring nodes by comparing the real network’s Cp to 100 random networks. The characteristic path length (Lp) represents the average minimum number of connections required to link any two nodes in the network. The normalized characteristic path length (*λ*) measures information transmission efficiency between nodes by comparing the real network’s Lp to 100 random networks. Node efficiency (NE) (*E*_local_) quantifies a node’s ability to communicate with other nodes in the network, while global efficiency (*E*_glob_) describes the overall information transfer efficiency of the network. The small-world index (*σ*) = *γ*/*λ*, with a value >1.1 indicating the small-world attribute of the network. Local properties include: DC, which is the degree to which a node is directly connected to the adjacent node in the network, reflecting the node’s information communication ability in the power network; Betweeness centrality (BC), is a measure of the connection ability of different nodes connected to a node, and describes its influence on the information flow between other nodes; NE indicates network node parallel information transfer efficiency; node local efficiency (NLE) refers to the communication efficiency between the first neighbour of the node when the node is removed. Additionally, to ensure a comprehensive assessment and avoid bias due to inter-group comparisons under a single sparse threshold, we calculated the area under curve (AUC) for each network indicator, offering a unified metric for the topological representation of the brain network across various threshold conditions.

#### NBS analysis

Using NBS v1.2 (https://www.nitrc.org/projects/nbs/) in the Matlab2016b to analyse NBS, one-sample *t*-test was used to compare functional connected networks between groups. Statistical differences between SLE and HCs groups were measured by permutations tests, in which years of education, cognitive status (MMSE scores), GMV, WMV, fame-wise displacement (FD) and medication were regressed as covariates. Specifically, the permutations distribution of the differences was calculated by randomly shutting the two groups of subjects 10 000 times and calculating the difference between the groups. Then, the *P*-value was obtained by calculating the percentage of the threshold *t*_0_-value (3.5) of the two samples after replacement that was greater than the true *t*-value, and *P* < 0.01 was the threshold of statistical significance. Here, NBS was used for multiple comparison correction to help define the interconnected significant subnetwork edges.^18,34^ BrainNet view was used to present subnetworks with significant differences between the two groups.

### Support vector machine classification based on NBS

NBS-predict is a prediction-based extension of the NBS method that combines ML and NBS features to identify graph components and their corresponding prediction accuracy, allowing researchers to extract meaningful information from large-scale neural network data characteristics. This is achieved by integrating advanced ML algorithms such as support vector machine (SVM) and decision trees within a nested cross-validation framework.^35^ Detailed analysis procedures are provided in the Supplementary materials.

### Statistical analysis

Analysis was performed with SPSS 24.0. The non-parametric K–S test assessed the measurement data’s normality. Skew distribution data were expressed as median (interquartile range), whereas normal distribution data were expressed as mean ± SD ($\bar{x}\pm s$). The two-independent sample *t*-test or the Mann–Whitney U rank sum test was used to compare the differences in measurement data between the two groups. The χ^2^ test compared the sex composition ratio of the two groups. The criterion for statistical significance was *P* < 0.05 (double tail). We utilized GRETNA’s Metric Comparison module to compare the AUC of each network indicator within the *D*_min_ – *D*_max_ range using a two-sample *t*-test, with years of education, cognitive status (MMSE scores), GMV, WMV, FD and medication as covariables. The AUC index is very sensitive to detect changes in brain functional topology, and can avoid bias caused by inter-group comparison under a single sparsity threshold. For global indicators, the criterion for statistical significance was *P* < 0.05. For local indicators, due to the number of severe nodes, false discovery rate (FDR) multiple comparisons with a threshold of *q* < 0.05 were used to adjust the results. Also calculated was Cohen’s *d* value to assess effect size. The correlation between clinical data and network indicators with aberrant comparisons between groups was analysed using GraphPad 8.0.2. The criterion for statistical significance was *P* < 0.05.

## Results

### Demographic and clinical data

MRI data was collected from 140 SLE patients and 111 HCs in this study. Some subjects were excluded due to partially absent image data (3 SLE and 2 HCs), structural abnormalities found by routine MRI (1 SLE and 1 HCs), excessive head movement (7 SLE and 5 HCs) and quality control failure (2 SLE and 1 HCs). The study finally included 127 SLE patients and 102 HCs. The SLE and HCs groups were matched in age (*t* = −0.831, *P* = 0.407) and sex (*χ*^2^ = 1.441, *P* = 0.230). Demographics and clinical data are shown in Table 1.

**Table 1:** Demographic and clinical data results of SLE group and HCs group

	SLE group (*n* = 127)	HCs group (*n* = 102)	*t*/*z*/χ^2^	*P*-value
Gender (female/male)	103/24	76/26	1.441	0.230
Age (year)	29.84 ± 7.00	30.63 ± 7.24	−0.831	0.407
Years of education	12.00 (9.00, 15.00)	15.00 (12.00, 16.00)	−5.363	0.000
Course of disease (month)	12.00 (3.00, 24.00)			
Treatments				
Drug-naive	24	0		
GCs alone	22	0		
GCs + HCQ	40	0		
GCs + ISA	41	0		
Use of psychoactive drugs	0	0		
Presence of neurological manifestations	0	0		
SLEDAI-2k	10.00 (6.00, 14.00)			
MMSE	28.00 (25.00, 29.00)	30.00 (30.00, 30.00)	−10.759	0.000
HAMA	7.00 (4.00, 11.00)	0.00 (0.00, 0.25)	−12.978	0.000
HAMD	8.00 (5.00, 13.00)	0.00 (0.00, 0.00)	−12.600	0.000
TIV (cm^3^)	1400.98 ± 113.49	1401.77 (1347.13, 1468.89)	−1.098	0.272
GMV (cm^3^)	628.22 ± 52.79	652.47 ± 52.03	−3.476	0.001
WMV (cm^3^)	473.64 ± 47.61	489.00 (452.27, 513.41)	−2.256	0.024

SLE, systemic lupus erythematosus; HCs, healthy controls; SLEDAI-2k, SLE disease activity index 2000; GCs, glucocorticoid; HCQ, hydroxychloroquine; CTX, cyclophosphamide; ISA, immunosuppressive agent (include motemycophenolate, methotrexate, leflunomide, cyclosporin, thalidomide and triptolide); MMSE, Mini-Mental State Examination; HAMA, Hamilton anxiety scale; HAMD, Hamilton depression scale; TIV, total intracranial volume; GMV, grey matter volume; WMV, white matter volume.

### Brain volume analysis

There was no significant difference in TIV between the two groups (*z* = −1.098, *P* = 0.272). However, GMV (*t* = −3.476, *P* = 0.001) and WMV (*z* = −2.256, *P* = 0.024) were significantly reduced in SLE compared with HCs (see Table 1).

### Functional connection network analysis based on graph theory

**Figure 1:**
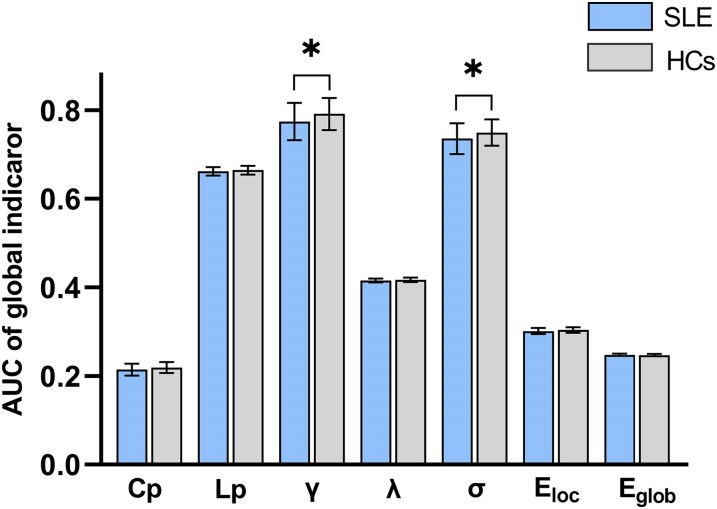
**Results of the between-group comparison of global indicators of functional connectivity networks in the SLE group and the HCs group.** Two-independent sample *t*-test found that *γ* and *σ* were significantly decreased in SLE group (*n* = 127) compare to HCs group (*n* = 102); AUC, area under the curve; Cp, clustering coefficient; Lp, characteristic path length; *γ*, normalized clustering coefficient; *λ*, normalized characteristic path length; *σ*, small-world index; *E*_loc_, local efficiency; *E*_glob_, global efficiency; SLE, systemic lupus erythematosus; HCs, healthy controls; Vertical bar chart: the mean value of AUC; Error bars: SD; *P < 0.05.

For global indicators, compared with the HCs group, the AUC values of *γ* (*t* = 2.1617, *P* = 0.0317, Cohen’s *d* = 0.4368), *σ* (*t* = 2.1050, *P* = 0.0364, Cohen's *d* = 0.4142) were significantly reduced in SLE groups. There was no significant difference in Cp (*t* = 1.1537, *P* = 0.2499, Cohen’s *d* = 0.3436) and, Lp (*t* = 1.1386, *P* = 0.2561, Cohen’s *d* = 0.2448), *λ* (*t* = 1.1782, *P* = 0.2300, Cohen’s d = 0.2553), *E*_glob_ (*t* = 1.1411, *P* = 0.2551, Cohen’s *d* = 0.2745) and *E*_loc_ (*t* = 1.3128, *P* = 0.1906, Cohen’s *d* = 0.3817) between the two groups (see Fig. 1). We have also conducted a statistical analysis of the inter-group differences in the global indicators mean values of the two groups of FC networks under different sparsity, and the results are shown in Supplementary Fig. 1 and Supplementary Table 1. In order to compare the results obtained by different network construction methods, we also calculated the weighted network. The results of the weighted network showed that there was no statistically significant difference in the global network between the two groups, but except for Lp, the inter-group difference trends of other global indicators were the same as the results of the binary network (see Supplementary Fig. 2). For local indicators including BC, DC, NE and NLE, the differences between the two groups were not significant after FDR correction.

### Correlation analysis between clinical data and abnormal network indicators

Spearman correlation analysis was performed on SLEDAI-2k, MMSE, HAMD, HAMA and AUC of aberrant global network indicators in the SLE group. No significant correlation was observed between the clinical data and the network indicators.

### NBS analysis

**Figure 2:**
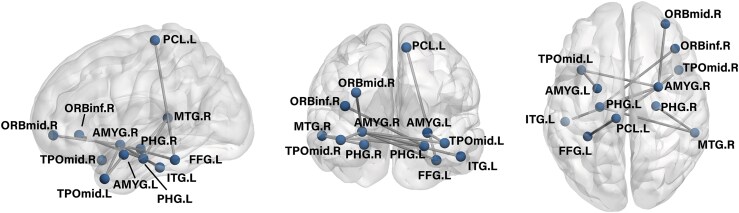
**Subnetworks with reduced connectivity in the SLE group.** Connections in the subnetwork identified by the whole-brain NBS method with threshold 3.5, where functional connectivity is significantly reduced in SLE group (*n* = 127) compare to HCs group (*n* = 102). Number of abnormal subnetworks: 1; Number of nodes: 12; Number of edges: 11; *P* = 0.024. A, sagittal; B, coronal; C, axial; L, left; R, right; PHG, ParaHippocampal gurys; AMYG, amygdala; TPOmid, temporal pole:middle temporal gyurs; PCL, paracentral lobule; ITG, inferior temporal gyrus; ORBmid, middle frontal gyrus, orbital; ORBinf, inferior frontal gyrus, orbital; MTG, middle temporal gyrus; FFG, fusiform gyrus; SLE, systemic lupus erythematosus; HCs, healthy controls; Two-independent sample *t*-test; *P* < 0.05.

The network connectivity of a subnetwork consisting of 12 nodes and 11 edges within the SLE group was found to be significantly lower than that of the HCs (threshold 3.5, *P* = 0.024), as illustrated in Fig. 2. The nodes of the subnetwork include bilateral AMYG, bilateral parahippocampal gyrus (PHG), bilateral temporal pole: middle temporal gyurs (TPOmid), left inferior temporal gyrus (ITG), left paracentral lobule (PCL), left fusiform gyrus (FFG), right middle frontal gyrus, orbital (ORBmid), right inferior frontal gyrus, orbital (ORBinf) and right middle temporal gyrus (MTG). It is noteworthy that none of the subnetworks in the SLE group exhibited increased FC than those in the HCs group.

## Discussion

The present study used BOLD-fMRI data to construct and compare FC networks between SLE and HCs patients to understand the network mechanism underlying brain dysfunction in SLE patients. We discovered the suboptimal state of the global network properties in the SLE group. NBS analysis of SLE patients revealed a subnetwork with much lower connectivity. We further investigated the applicability of the SVM classifier for SLE and HCs classification using functional NBS features for the first time, but the classification performance was poor (see Supplementary Fig. 3 and Supplementary Table 2). These findings enhanced our understanding of the neuropathological processes involved in the alteration of brain networks in SLE patients.

The small-world attribute symbolizes the brain’s balance between functional integration and separation, which ensures regular information processing and lowers network maintenance costs. The small-world attribute is stronger the higher the *σ* value.^17,36^ Patients with SLE showed a significant reduction in *σ* in this study. This suggests that SLE patients’ brain FC network may be impaired by disease since it is not optimal like HCs’. Cp and *γ* indicate the degree of interconnection between network nodes and adjacent nodes and are important indicators reflecting the ability of network function separation. Specifically, Cp represents the clustering coefficient, which measures the tendency of nodes to form tightly connected groups, while *γ* reflects the balance between local specialization and global integration within the network. The decrease of *γ* in SLE patients reflects the inefficiency of information interaction in closely connected brain regions, that is, the ability of information specialization is weak at the global level. This inefficiency may contribute to cognitive deficits and mood disorders commonly observed in SLE patients, as specialized processing in certain brain regions is crucial for higher-order functions. Similar results have been reported in patients with end-stage renal disease with cognitive impairment and in patients with low-back pain.^37,38^ These similarities suggest that *γ* may be a sensitive marker of brain function to detect network dysfunction in patients with multiple neuropsychiatric disorders and help us understand the mechanisms of brain functional network impairment in disease-related neurofunctional abnormalities. Lp and *λ* describe the efficiency of information transmission between nodes and are important indicators reflecting the integration ability of network functions. The smaller Lp and *λ* are, the faster the information transmission speed of the network. The results of this study suggest that there is no significant impairment in the functional integration of brain functional networks in SLE patients. On the contrary, the two functional integration indexes of Lp and *λ* in SLE group are better than those in HCs group, but the difference between groups is not significant. This discovery suggests that both compensatory and damaging factors may coexist within the FC networks of the brain in SLE. According to graph theory, the ratio *σ* = *γ*/*λ*, this study observed a significant reduction in *σ* and *γ* values among SLE patients. However, the reduction of *λ* was not found to be significant, indicating that the destructive decrease in *γ* is more pronounced than the compensatory decrease in *λ*, ultimately resulting in a decompensation of *σ*. This alteration in the functional network primarily presents as decompensation, which may represent one of the mechanisms affecting the brain network in SLE.

No significant abnormalities were found in the local indicators of global brain FC in the SLE group. This lack of significant findings may be attributed to changes in the global properties of the brain FC network in SLE, which may involve generic nodes and not be readily apparent at the individual node level. As a result, these local changes could not be detected after rigorous correction in this study. Additionally, the observed findings may also be influenced by the sample selection in this study, and further validation is required in future research.

The results of this study differ from prior reports of brain FC networks in SLE patients. Cao *et al*.^35^ comprised HCs and 41 SLE patients. They also performed graph theory-based network analysis and built each participant’s whole-brain FC network using the AAL-116 template. The study found no significant differences between the two groups in global indices (*σ*, *γ*, *λ*, *E*_glob_ and *E*_glob_). Local network indicators showed that the SLE group had significantly lower *E*_nodal_ in the right insula, bilateral putamen and bilateral transverse temporal gyrus, as well as lower nodal degree in the right AMYG and bilateral transverse temporal gyrus than the HCs. The study found a correlation between MMSE, disease duration and network indicators in certain brain regions.^19^ MRI parameters and sample selection may explain the heterogeneity in the two research results. The study with a relatively small sample size did not specifically exclude patients with SLE who had neuropsychiatric symptoms, while our study, which included more patients, did not include patients with NPSLE. There are few researches on brain functional network abnormalities in SLE patients and future multi-centre, large-sample studies and studies targeting SLE patients with different clinical characteristics are still needed to verify and clarify.

Intact brain structure is the basis for normal brain function. When investigating changes in brain function among SLE patients, it is essential to account for the potential influence of structural alterations on the findings. To minimize bias arising from structural differences during FC comparisons between groups, GMV and WMV were included as covariates in all inter-group analyses. We discovered that SLE patients had significant decreases in WMV and GMV. In other words, loss of brain parenchyma is a feature of brain injury in SLE patients in addition to alterations in brain functional networks. This is consistent with previous reports. A prospective study with a small sample found that SLE patients had decreased GMV in brain areas such as the bilateral temporal poles, left cerebellum and left insula early in the course of the disease (within 5 months of diagnosis). When reaching low disease activity, additional areas with lower GMV were found in the SLE group in the right hemisphere cuneus/precuneus, MTG and other brain regions. The WMV of the bilateral superior longitudinal fasciculus, corticospinal tract, cingulate gyrus and inferior frontal–occipital fasciculus was lower in lupus patients than in HC patients.^39^ Previous studies by our research group have also found that GMV and WMV in the SLE group were significantly reduced, and immunosuppressive treatment has a protective effect on brain atrophy in SLE.^40^ These findings suggest that changes in brain structure and function may jointly affect SLE patients, and the relationship between the two needs to be further clarified by a series of studies such as structural and functional coupling or mediation effect analysis.

Using NBS, the whole-brain FC network of SLE patients was found to have a subnetwork with significantly reduced connectivity in this study. Twelve nodes and eleven connected edges made up this subnetwork. As shown in Fig. 2, the left PHG and the right AMYG are at the centre of the subnetworks with reduced FC in SLE because they are implicated in the greatest number of abnormal connections. The AMYG and PHG, as part of the limbic system, play a crucial role in the development of mood disorders. It is intricately connected to various cortical and subcortical areas through fibre bundles, forming a complex neural circuit system that is essential for processing emotions.^41^ Temporal pole and ITG were found to be associated with multiple higher cognitive functions, such as visual recognition, language comprehension, decision-making and emotion regulation.^42,43^ Furthermore, the medial temporal pole area is a key component of the AMYG in the limbic system, responsible for integrating external-sensory information with internal-sensory states based on social interactions.^44^ In this study, it is found that there is an obvious functional disconnection between these temporal lobe nodes and the limbic system. The limbic system is located on the medial side of the temporal lobe, and in addition to their close anatomical relationship, the two systems share functional synergy. The temporal lobe is involved in the formation of emotional memories and regulation of emotions through interaction with other parts of the limbic system, such as the AMYG. The PCL is the medial side of the frontal lobe, a continuing part of the anterior and posterior central gyrus. It serves as one of the cortical centres for motor control, primarily regulating sensation, motor functions and urination in the contralateral lower limbs^45,46^. Additionally, it is recognized as a highly connected node within the structural core of the cerebral cortex, forming dense, short connections with regions associated with the DMN.^47^ While some studies suggest this area may play a role in broader emotional processes, strong evidence directly linking it to emotional functioning remains limited.^48,49^ The structure of the FFG located on the basal surface of the temporal and occipital lobes is a key brain region responsible for processing visual information, especially the recognition of faces and objects.^50^ Disconnection of these two brain regions is the first time it has been observed in SLE patients and may lead to potential impairment of visual and motor control functions, and more studies are needed to confirm it. There is an evidence that neuronal activity in the orbital frontal cortex and the AMYG, as well as connections between these regions, contribute to social decision-making.^51^ Our findings indicate alterations in functional brain connectivity networks in SLE patients. However, it is crucial to note that these results do not directly link atypical functional connectivity to specific cognitive or emotional impairments. Although neuropsychiatric symptoms are prevalent in a subset of patients with SLE, the observed changes in connectivity may reflect broader neural adaptation or compensatory mechanisms rather than explicit pathological processes associated with neuropsychiatric manifestations. Future research is needed in combination with neuropsychological assessment and longitudinal design to explore potential relationships between altered functional networks and clinical symptoms.

The present study discovered that SLE patients may have weakened FC between emotion- and cognition-related brain regions before exhibiting obvious neuropsychiatric symptoms. This is consistent with the decreased network connection observed in global network studies and could be the brain network basis for related symptoms in SLE patients. Prior research has identified abnormal brain functional network connectivity in SLE patients. For instance, Nystedt discovered decreased connectivity in the DMN, the central executive network (CEN) and between the DMN and CEN in SLE patients. Conversely, heightened connectivity was predominantly observed within and between the SMN in SLE patients compared to HCs. Furthermore, when comparing SLE patients with and without neuropsychiatric symptoms, it was found that NPSLE patients exhibited more pronounced hypoconnectivity.^22^ These findings suggest a potential link between altered FC and neuropsychiatric manifestations in SLE, highlighting the need for further investigation into the underlying mechanisms and potential therapeutic implications.

Previous studies in ML focused on brain function indicators in SLE have demonstrated superior performance compared to the current study^52,53^ (see Supplementary Fig. 3 and Supplementary Table 2). Predictor factors’ biological significance and contribution remain challenging to measure. For two reasons, we used whole-brain FC and ML feature selection and/or classification in this investigation. We used the NBS approach and ML to evaluate whole-brain FC classification in SLE, unlike earlier research that used isolated or scattered characteristics as predictive variables. This helped us to find more biologically relevant brain function biomarkers. Second, our method derives feature contribution weights, making the network more interpretable. We must emphasize here that despite the use of feature selection methods, and despite the presence of significantly different subnetworks between groups, unexpected and unaccounted structural differences contributed to the poor classification results in this study. This finding highlights the dataset’s complexity and suggests that additional methodological enhancements, such as multimodal data fusion, may be required to get more robust classification results for SLE patients.

Several caveats should temper the interpretation of our findings. First, while we matched control and SLE patients on key demographic variables, potential selection bias remains, and subtle differences in unmeasured factors (e.g. socioeconomic status and subclinical vascular risk) may affect brain measures. Second, the cross-sectional design precluded definitive conclusions about whether the observed atrophy and functional changes were caused by chronic disease or accumulated by chance during an episode. A previous longitudinal study has demonstrated that the destruction of the white matter microstructure of the brain increases significantly with the course of SLE,^54^ but prospective data on brain atrophy in SLE are lacking to date. Crucially, the persistence of functional network alterations after controlling for structural covariates means that SLE-related neurological dysfunction cannot be completely reduced to structural atrophy—a finding consistent with Su *et al*., which showed both grey matter volume atrophy and functional abnormalities in NPSLE brain tissue. However, the affected areas of the brain do not overlap.^53^ In future studies, the combination of multimodal sequential imaging, longitudinal imaging studies and structure–function coupling studies will help clarify the temporal dynamics of brain injury in SLE and the relationship between brain structural and functional injury.

This study has limitations: First, the 1.5T fMRI used in this study may miss some subtle signal changes due to its low signal-to-noise ratio. To mitigate some of its inherent limitations, this study carried out strict quality control on MRI data quality at the image acquisition stage, carefully examined the images of each participant and eliminated eight cases of incomplete scanning and poor-quality data. In the data analysis phase, we eliminated the data of 12 subjects with excessive head movement amplitude and returned the noisy signal. In the statistical analysis stage, we included the head motion parameters in the covariate. Future research using higher field strengths could potentially provide a more comprehensive understanding of the brain networks in SLE patients. Second, the definition of network nodes is crucial to the study of brainnetome, but only AAL-116 was used in this study, and different templates with finer division of brain regions need to be added in the future to verify the repeatability of this study. Third, the present study's cross-sectional design necessitates future longitudinal research to complement it to evaluate the dynamic alterations in functional networks brought on by SLE. Fourth, we included participants who had received treatment with medications such as glucocorticoids and immunosuppressants. However, the mechanisms of action of these medications are intricate and may have potential effects on haemodynamics. To address this, we have incorporated medication treatment as a covariate in our statistical regression analysis. It is important to recognize that while this approach helps to control for the potential effects of medication, the precise impact of these drugs on brain networks and haemodynamics remains a limitation of our study. Eventually, research is necessary to fully comprehend the implications of medication treatment on our findings. Fourth, we used the MNI template for registration and standardization in our study, mainly because it is widely used in brain imaging studies and has good reproducibility and comparability. However, we are also aware that there may be anatomical differences between different populations. For example, Jao *et al*.^55^ have developed the NTU-CBT template based on the MNI template for the Chinese population, which has good potential in analysing the morphological and functional characteristics of the Chinese population. We will consider exploring more templates suitable for the Chinese population in subsequent studies to improve the accuracy and relevance of the results.

## Conclusion

SLE patients showed impaired global FC network topology, characterized by significant disruption of functional separation, reduced small-world properties and a subnetwork with abnormally reduced connectivity.

## Supplementary Material

fcaf130_Supplementary_Data

## Data Availability

The datasets generated and analysed during the current study are not publicly available due to protection of individuals’ privacy but are available from the corresponding author on reasonable request.
