# Supplementary material for: Abnormal brain functional networks in systemic lupus erythematosus: a graph theory, network-based statistic and machine learning study
Source: Brain Commun. 2025 Apr 2;7(2):fcaf130. doi: 10.1093/braincomms/fcaf130 (PMC11979335; doi:10.1093/braincomms/fcaf130)
Supplement: fcaf130_Supplementary_Data [file fcaf130_supplementary_data.docx]

**Process of structural magnetic resonance preprocessing and calculation of total intracranial volume, gray matter and white matter volume**

In SPM12 (statistical parametric mapping software, http: // www.fil.ion.ucl.ac.uk/spm) running CAT12 tool box (computational anatomy toolbox, version 12.7) using the default parameters for T1 structure as data processing and analysis.

1. spatially normalized using the DARTEL algorithm

The 3D-T1-weighted brain structure images of all subjects were registered to the MNI (Montreal Neurological Institute) standard space previously combined as the initial location, and the standardized segment voxel size was 1.5mm^3^.

2. segment

The standardized brain structure image was effectively segmented into gray matter, white matter and cerebrospinal fluid. Following segmentation, each tissue class was warped to a template image using the high dimension DARTEL normalization method. The tissue class images were modulated during DARTEL normalization to preserve the original volumes of the tissue class.

3.Smooth

8mm full-width at half-maximum (FWHM) Gaussian kernel is used to smooth the gray image after segmentation and modulation, and the smoothed image data will be more in line with normal distribution.

The volume of gray matter, white matter and cerebrospinal fluid of each subject was calculated by using CAT12 tool box.

**Support vector machine (SVM) classification based on NBS**

The NBS-Predict 1.0.0-beta.5 (https://www.nitrc.org/projects/nbspredict/) is used based on the Matlab 2019 platform to perform ML predictions based on the brain functional connection network. ^1^

The specific steps include: (1) correlation matrix input; (2) Input of brain regions (nodes): This study defined nodes according to the AAL-116 template; (3) Model building: The comparison matrix of the two groups was designed, and the SVM classification model was selected for the ML model; (4) Advanced options: Set the cross-validation as 10-fold cross-validation (CV), that is, the data is divided into 10 subsets, each iteration, select one subset as the test set, and the remaining subsets are combined as the training set. Repeat 10 times to reduce variation in model performance estimates, and seed to 42. The edges with a *P* ＜ 0.01 were selected for further analysis; (5) Years of education, TIV, FD, and medication were regressed as covariates; (6) Run the model and output the model's performance indicators, ring network and disconnected subnetwork visualization results. ^2^

**The results of SVM classification based on NBS**

**Classifier performance evaluation results**

The brain FC network was used as a feature in the NBS-based SVM classification of the SLE and HCs groups. AUC was 0.607, accuracy 0.608, sensitivity 0.594, specificity 0.620, recall 0.594, and F1 value 0.578 for the classification.

**The most effective subnetworks for SLE classification**

The weight of a subnetwork in NBS-based SVM classification represents its model contribution. We constructed disconnected subnetworks using the most conservative weight threshold (1.0) for the SLE group to better assess their neuroimaging biomarker efficacy in SLE classification. As shown in Supplementary Figure. 3 and Supplementary Table 2, a misconnected SLE subnetwork with 22 nodes and 29 connected edges was detected when the weight threshold was set to 1.0.


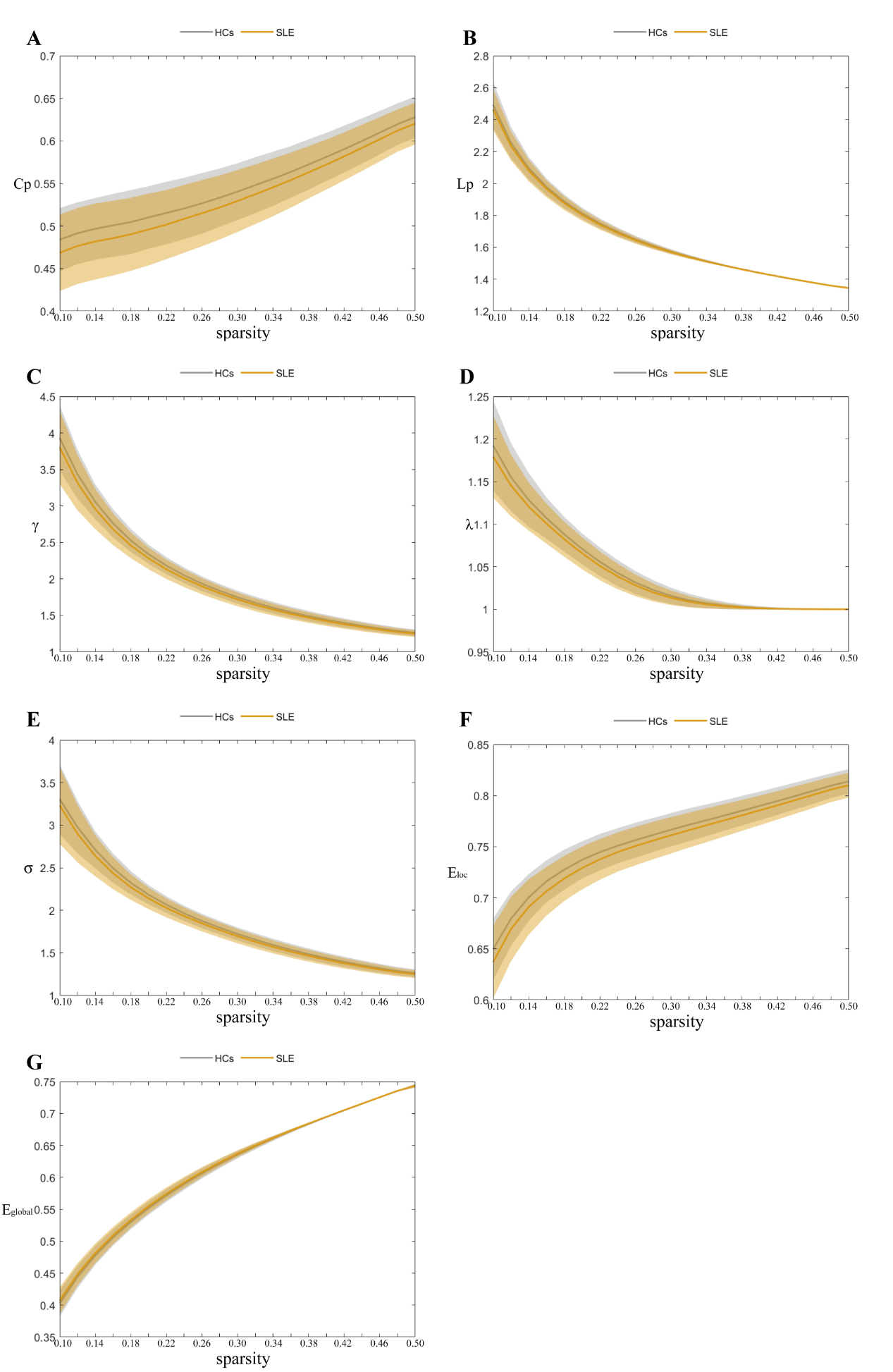


Supplementary Figure. 1. The mean and standard deviation of each sparsity of the two groups for each global functional connection indicators in SLE and HCs group

A-G Curves displaying the mean and standard deviation of each sparsity of the two groups for each global functional connection indicators in SLE group (n = 127) and HCs group (n = 102). Colored error bars indicate the SD of topology indicators across sparsities (0.10 ~ 0.50 with a 0.02 interval). Cp: clustering coefficient; Lp: characteristic path length; γ: normalized clustering coefficient; λ: normalized characteristic path length; σ: small-world index; E_loc_: local efficiency; E_glob_: global efficiency; SLE, systemic lupus erythematosus; HCs, healthy controls.


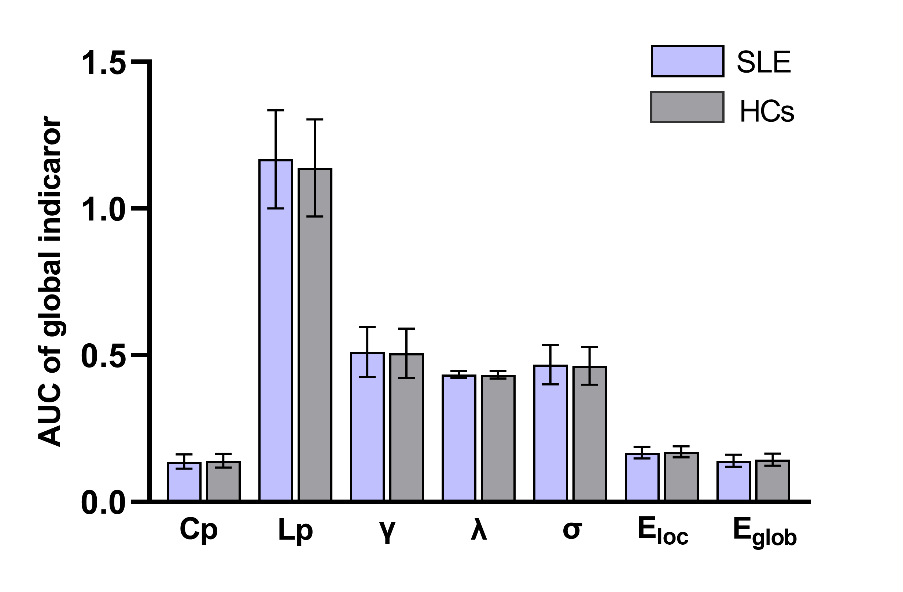


Supplementary Figure. 2 Results of the between-group comparison of global indicators of functional connectivity weighted networks in the SLE group and the HCs group.

Two independent samples *t* test found that there were no significant differences (*P*＞0.05) in all global indicators between the SLE group (n = 127) and the HCs group (n = 102). AUC: area under the curve; Cp: clustering coefficient; Lp: characteristic path length; γ: normalized clustering coefficient; λ: normalized characteristic path length; σ: small-world index; E_loc_: local efficiency; E_global_: global efficiency; SLE, systemic lupus erythematosus; HCs, healthy controls; Vertical bar chart: the mean value of AUC (area under the curve); Error bars: standard deviation (SD).


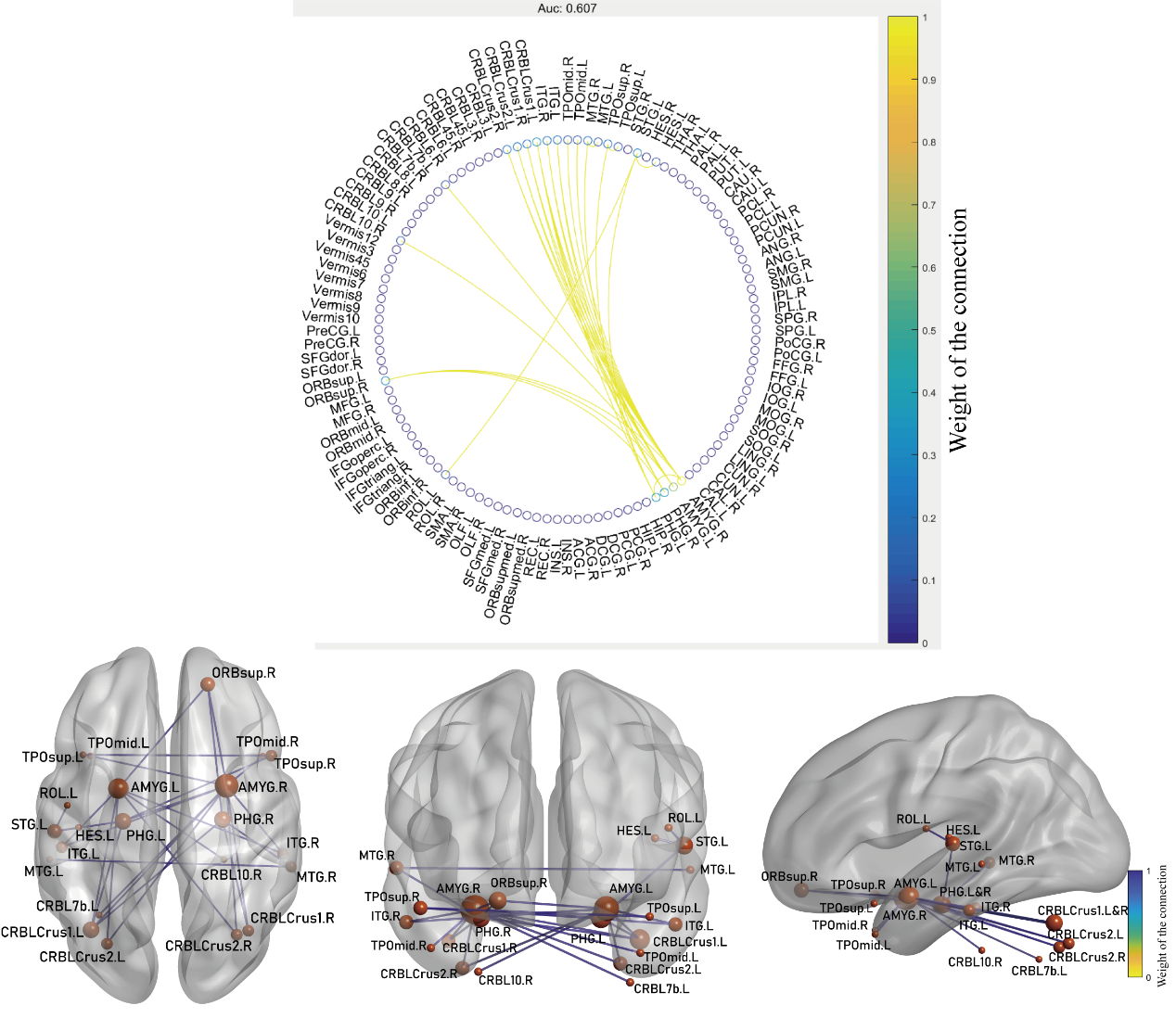


Supplementary Figure. 3 Disconnected subnetworks of SLE when the classification contribution weight is 1

The ring network diagram (above) and the misconnected sub-network (below) of SLE (n = 127) with the classification contribution weight threshold of 1, including 22 nodes and 29 connected edges. The color bar represents the weight of each connected edge and node, and the size of the node sphere represents the size of the nodal degree. L: left R: right; AMYG: Amygdala; PHG: ParaHippocampal gurys; CRBLCrus: Cerebelum_Crus; ORBsupmed: Superior frontal gyrus, medial orbital; STG: Superior temporal gyrus; TPOsup: Temporal pole: superior temporal gyrus; MTG: Middle temporal gyrus; ITG: Inferior temporal gyrus; ROL: Rolandic operculum; HES: Heschl gyrus; TPOmid: Temporal pole: middle temporal gyrus. The heat-map scale bar represents the weight of the connection.

Supplementary Table 1 *P*-values of global indicators statistical comparisons between SLE (n = 127) patients and HCs (n = 102) with two-sample *t*-tests

| Sparsity | *P* (Cp) | *P* (Lp) | *P* (γ) | *P* (λ) | *P* (σ) | *P* (E_loc_) | *P* (E_global_) |
| --- | --- | --- | --- | --- | --- | --- | --- |
| 0.10 | 0.006 | 0.110 | 0.086 | 0.056 | 0.199 | 0.005 | 0.090 |
| 0.12 | 0.007 | 0.090 | 0.042 | 0.051 | 0.073 | 0.051 | 0.075 |
| 0.14 | 0.06 | 0.105 | 0.017 | 0.051 | 0.022 | 0.006 | 0.087 |
| 0.16 | 0.008 | 0.078 | 0.013 | 0.040 | 0.013 | 0.001 | 0.066 |
| 0.18 | 0.009 | 0.054 | 0.010 | 0.036 | 0.008 | 0.002 | 0.046 |
| 0.20 | 0.017 | 0.041 | 0.011 | 0.034 | 0.008 | 0.003 | 0.036 |
| 0.22 | 0.021 | 0.036 | 0.010 | 0.033 | 0.006 | 0.006 | 0.033 |
| 0.24 | 0.018 | 0.047 | 0.019 | 0.047 | 0.012 | 0.012 | 0.043 |
| 0.26 | 0.019 | 0.057 | 0.031 | 0.055 | 0.017 | 0.018 | 0.054 |
| 0.28 | 0.019 | 0.049 | 0.029 | 0.048 | 0.014 | 0.019 | 0.047 |
| 0.30 | 0.023 | 0.055 | 0.035 | 0.054 | 0.017 | 0.018 | 0.053 |
| 0.32 | 0.022 | 0.086 | 0.028 | 0.085 | 0.013 | 0.017 | 0.084 |
| 0.34 | 0.023 | 0.084 | 0.040 | 0.084 | 0.022 | 0.023 | 0.083 |
| 0.36 | 0.020 | 0.110 | 0.035 | 0.109 | 0.020 | 0.023 | 0.109 |
| 0.38 | 0.022 | 0.111 | 0.044 | 0.111 | 0.027 | 0.023 | 0.111 |
| 0.40 | 0.025 | 0.071 | 0.043 | 0.071 | 0.028 | 0.020 | 0.071 |
| 0.42 | 0.022 | 0.063 | 0.048 | 0.063 | 0.033 | 0.021 | 0.063 |
| 0.44 | 0.021 | 0.093 | 0.053 | 0.093 | 0.038 | 0.025 | 0.093 |
| 0.46 | 0.022 | 0.087 | 0.053 | 0.087 | 0.039 | 0.022 | 0.087 |
| 0.48 | 0.021 | 0.241 | 0.043 | 0.108 | 0.033 | 0.021 | 0.241 |
| 0.50 | 0.022 | 0.662 | 0.077 | 0.057 | 0.049 | 0.022 | 0.657 |

Cp: clustering coefficient; Lp: characteristic path length; γ: normalized clustering coefficient; λ: normalized characteristic path length; σ: small-world index; E_loc_: local efficiency; E_global_: global efficiency; SLE, systemic lupus erythematosus; HCs, healthy controls.

Supplementary Table 2 The nodes contained in subnetworks under the maximum category classification contribution weight thresholds

| Node | Nodal Degree | Node | Nodal Degree |
| --- | --- | --- | --- |
| Right Amygdala | 11 | Right Cerebelum_Crus1 | 2 |
| Left Amygdala | 7 | Left Cerebelum_Crus2 | 2 |
| Left Parahippocampal gyrus | 4 | Right Cerebelum_Crus2 | 2 |
| Right Parahippocampal gyrus | 4 | Left Rolandic operculum | 1 |
| Left Cerebelum_Crus1 | 4 | Left Heschl gyrus | 1 |
| Right Superior frontal gyrus, medial orbital | 3 | Left Temporal pole: superior temporal gyrus | 1 |
| Left Superior temporal gyrus | 3 | Left Middle temporal gyrus | 1 |
| Right Temporal pole: superior temporal gyrus | 2 | Left Temporal pole: middle temporal gyrus | 1 |
| Right Middle temporal gyrus | 2 | Right Temporal pole: middle temporal gyrus | 1 |
| Left Inferior temporal gyrus | 2 | Left Cerebelum_7b | 1 |
| Right Inferior temporal gyrus | 2 | Right Cerebelum_10 | 1 |

References

1. Serin E, Zalesky A, Matory A, Walter H, Kruschwitz JD. NBS-Predict: A prediction-based extension of the network-based statistic. *NeuroImage.* 2021;244:118625.

2. Xia M, Wang J, He Y. BrainNet Viewer: a network visualization tool for human brain connectomics. *PloS one.* 2013;8(7):e68910.
